# Effect of topography and protecting barriers on revegetation of sandy land, Southern Tibetan Plateau

**DOI:** 10.1038/s41598-019-43034-8

**Published:** 2019-04-24

**Authors:** Chengrui Liao, Beichen Liu, Yannan Xu, Yingkui Li, Haidong Li

**Affiliations:** 1 Nanjing Forestry University, Nanjing, 210037 China; 2 Nanjing Institute of Environmental Sciences, Ministry of Ecology and Environment, Nanjing, 210042 China; 3 Centre of Co-Innovation for Sustainable Forestry in Southern China, Nanjing Forestry University, Nanjing, 210037 China; 4 School of Forestry and Environmental Studies, Yale University, New Haven, CT 06511 USA; 5 Department of Geography, University of Tennessee, Knoxville, TN 37996 USA

**Keywords:** Environmental impact, Environmental impact, Restoration ecology, Restoration ecology

## Abstract

Revegetation on sandy land has attracted worldwide attention, especially on the extremely fragile alpine eco-region of the Tibetan Plateau. However, the effectiveness of revegetation and its controlling factors have rarely been reported. We collected plant growths and species composition from seven field sites in 2011 and conducted a follow-up random investigation in 2016. The indicators, including richness and diversity, were used to compare the differences among these sites based on redundancy and cluster analyses. The results indicated that plant growth has different characteristics in different land types. The distribution and growth of *Artemisia sphaerocephala*, *Artemisia younghusbandii* and *Heteropappus gouldii* varied with topography, and the crown widths of *A. sphaerocephala* were 100.6 cm × 87.2 cm on barchan dune and 26.0 cm × 25.4 cm on moving sandy land at valley slopes. These species are likely the pioneer plants for revegetation on sandy land. It seems that sand-protecting barriers play an important role in revegetation. The stone and plastic checkerboard barriers increase plant diversity, while straw barrier promotes the plant growth. These findings provide useful guidance to the ongoing vegetation recovery on sandy land, an important component of the Project on Construction and Protection of Ecological Security Barriers on the Tibetan Plateau.

## Introduction

Desertification has become a worldwide concern because of its adverse impacts, such as the destruction of infrastructure, damage to economic loss, and increase in regional poverty and social instability^1,2^. Based on the Millennium Ecosystem Assessment (MA) in 2000, one quarter of land have been degraded in the world, while the impacts of socio-economic and policy drivers on land degradation have not been reduced since the United Nations Convention to Combat Desertification (UNCCD) entered into force^3^. Many countries have joined the UNCCD and made positive progress in desertification control^4^. For example, a series of projects have been launched against desertification in the Mediterranean countries of Europe^5–7^. In western Asia, including Iran and Iraq, extensive efforts also have been directed to combat desertification^8–11^. However, many projects have not been effective due to the lack of ecological function perspective, such as improper seeding, unsuitable sand barrier and unreasonable erosion control^12^.

The Tibetan Plateau, referred to as the “Third Pole” of Earth and the “Water Tower of Asia”, is naturally dominated by aeolian processes, particularly wind erosion and dust emissions^2,13^. The area of sandification, a major type of desertification, reached 34.04 × 10^4^ km^2^ on the Tibetan Plateau by 2014^14^. In China, national scale and billion-dollar projects have been undertaken for desertification control^15,16^. Since 1999, Chinese government has implemented the “Western Regions Development Strategy” with a high priority to protect ecosystems on the Tibetan Plateau. A series of ecological protection and restoration projects have been conducted to prevent vegetation loss and recover degraded land and its related ecological services^17^. This effort includes planting artificial forest and shrubs over the plateau^18,19^. Revegetation on sandy land is a crucial mission for people living in arid, semi-arid, and dry subhumid areas^20^. Previous reports show that revegetation includes not only vegetation coverage, but also recovery of community structure^21,22^ and plant diversity^23,24^. The planting of shrubs, trees^25^, and grazing exclusion^26,27^ has a positive impact on revegetation. However, few studies have focused on the effectiveness of recovery techniques^20^ and the relationship between vegetation and topography^28,29^. It is important to identify the key limiting factors, such as sandy land types and sand-protecting barriers, and how these factors affect the revegetation.

Revegetation on sandy land is limited by surrounding environment^30–32^. Habitat factors on sand dune are easily changed by wind erosion, sand accumulation, and dune encroachment; thus plant distributions are limited by their positions^20^. The micro-topography of sandy land affects the plant distribution, but it can be changed by protecting barriers. In 2008, we established a set of experimental plots with no irrigation systems to observe plant species growth, habitat factors, the best seeding period, as well as the pattern and structure of native psammophytes population. However, revegetation patterns on different sandy land types and the influences of topography are still poorly understood due to the adverse effect of mountainous environment and harsh climate conditions for field investigation. This paper provides our first investigation on the vegetation coverage, crown width, and species composition from different sandy land types and protecting barriers. We also analyzed the growth of plant species and vegetation community on different land types, identified the corresponding dominant species for each land type, and elucidated the effectiveness of sand-protecting barriers on revegetation. Finally, we summarized and put forward the patterns of revegetation and recommendations for the future development on the revegetation work of local government and residents. Specifically, the following questions were addressed: (1) is topography one of the key limiting factors that affect plant growth and distribution on different sandy land types? and (2) how does revegetation effectiveness change among sand-protecting barriers?

## Methods

### Study area

The Yarlung Zangbo River in China, stretching across the southern edge of the Tibetan Plateau from west to east, is approximately 2057 km long with a drainage area of 2.4 × 10^5^ km^2^ ^33^. The middle reaches of the Yarlung Zangbo River (Shigatse and Shannan wide valleys) are part of the alpine eco-region of the Tibetan Plateau with extremely fragile environment dominated by wind erosion, debris flow, landslide and soil salinization.

Three field sites were selected in Shannan and Shigatse wide valleys. The climate conditions and plant composition are shown in Table 1. The soil types are mainly aeolian sandy soil with coarse texture. The capacity of soil water and nutrients conservation is poor^34^. Field seeding experiments on sandy land were conducted in 2008 and 2009. The main testing plant species included the northern China’s psammophyte plant, such as *Artemisia sphaerocephala*, *Hedysarum scoparium* and *Calligonum mongolicum*, and the Tibet’s native species, such as *Sophora moorcroftiana* and *Artemisia wellbyi*. The growth of northern China’s psammophyte plants is better than that of Tibet’s native species^34^.

**Table 1:** climate conditions and plant composition of study area.

Study area	Climate type	Annual rainfall (mm)	Annual mean temperature (°C)	Surrounding plants
Shannan Wide Valley	Semi-arid	300–450	6.3–8.7	*S. moorcroftiana*, *A. wellbyi*, *Artemisia younghusbandii*, *Oxytropis sericopetala*, *Orinus thoroldii*, *Trikeraia hookeri*, *Salix xizangensis*, *Populus alba* var. *pyramidalis* and *Ulmus pumila*.
Shigatse Wide Valley	Warm and semi-arid	290–440	5.0–6.5	*S. moorcroftiana*, *Artemisia xigazeensis*, *A. wellbyi*, *O. sericopetala*, *O. thoroldii*, *T. hookeri*, *S. xizangensis*, *P. alba* var. *pyramidalis* and *U. pumila*

### Experimental design

**Figure 1:**
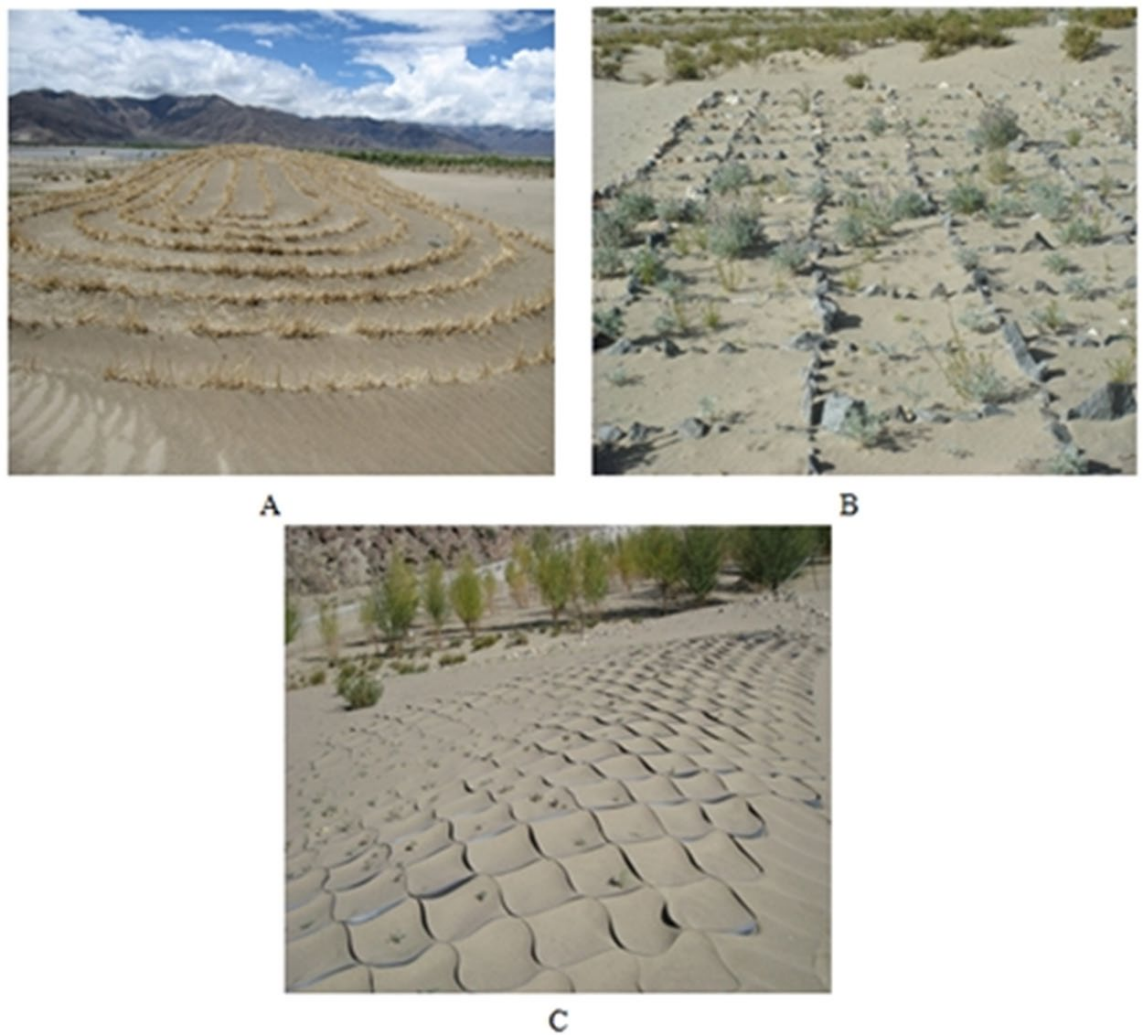
Sand-protecting barriers in moving sandy land on flood plain in 2009. (**A**) Straw barriers, (**B**) stone checkerboard barriers, (**C**) plastic checkerboard barriers.

We selected four types of sandy land topography to collect plant growth data, including Low sand belt (A), Sandy gravel ground (B), Barchan dune (C), and Moving sandy land on valley-slope (D). The sample selection and layout are shown in Table 2. The sand-protecting barriers are difficult to implement for high and cold sandy land, especially for the moving sandy land on valley-slope. Therefore, three sand-protecting barriers were only laid on moving sandy land on the floodplain, which was one of the four types, in 2008. The three sand-protecting barriers include straw barriers (D1 & D2), plastic and stone checkerboard barriers (D3), and untreated (Low sand belt, D4) (Table 3). Among them, D1 was near the river and D2 was close to the road (Fig. 1A). For D3, stone checkerboard barriers were used on downhill and plastic checkerboard barriers were used on uphill (Fig. 1B,C). Straw barriers were placed on D1 and D2 at intervals of about 1 m. The layer thickness and height were 10–20 cm and 30–40 cm, respectively (Fig. 1A). Each stone checkerboard barrier was 1 m × 1 m, the thickness and height were 10–20 cm and 20–30 cm, respectively (Fig. 1B). Each plastic checkerboard barrier was 25 cm × 25 cm, the height was 5 cm, and the material was mainly high-density HDPE (Fig. 1C).

**Table 2:** Field site situation of different sandy land topography.

Identifier	Location	GPS	Dune shape	Dune height/m	Dune density/%	Slope/(°)	Aspect	Soil particle composition	The number of sampling lines	The number of quadrats
A	Moving sandy land on flood plain	29°20′N, 90°54′E	Low sand belt	<1	<50	1–15	From north to south	Very fine sand	2	7
B	Moving sandy land on flood plain	29°20′N, 90°54′E	Sandy gravel ground	<1	<50	1–8	—	Very fine sand	2	8
C	Moving sandy land on river bank	29°20′N, 89°21′E	Barchan dune	2–5	60–70	1–18	From north to south	Very fine sand	—	6
D	Moving sandy land on valley-slope	29°20′N, 90°53′E	Moving sandy land on valley-slope	—	>80	1–31	Sutheast	Very fine sand	4	7

**Table 3:** Sample data of different sand-protecting barriers in moving sandy land on the floodplain in Shannan Wide Valley.

Identifier	The number of transects	The number of quadrats	Vegetation coverage/%
D1	2	12	12.83 ± 2.67 a
D2	2	20	14.39 ± 3.22 a
D3	2	16	19.06 ± 2.97 a
D4	2	8	19.64 ± 4.63 a

Vegetation coverage with significant differences are given as mean ± SE. Values with the same letter are not significantly (*P* > 0.05).

### Data collection

#### Soil sampling

In 2011, quadrats of 5 × 5 m were placed at each site along the sampling line on each sandy land type. For moving sandy land on valley-slope, the sampling line was laid on the southeast slope which was leeward slope and good for plant growth. The number of quadrats for each site is shown in Table 3. The basic situation is shown in Table 3 for sand-protecting barriers. Site variables included GPS locations, dune shape, dune height, dune density, slope, aspect, and soil particle composition. Dune density was determined by the rate of deposition of sand from a container with a specified size and number of holes into another box^35^. Soil particle composition was determined by the pipette method in a sedimentation cylinder, using sodium hexametaphosphate as the dispersing agent. In July 2016, the status of revegetation in these sites was examined using the random sampling method to compare the effect with that in 2011.

Soil samples (0–40 cm depth) were taken at five points in each quadrat in the four types of sandy land topography, and then bulked and well-mixed to make one composite sample (Table 2). After air drying, each composite sample was sieved to <2 mm for the analysis of soil particle composition, and to <0.1 mm for the determination of soil pH, organic matter, total nitrogen (N), available phosphorus (P) and available potassium (K).

The soil pH was determined in 1:2.5 soil-water extracts with a digital pH meter (Cyberscan 2100 model; Beijing, China). The organic matter was determined using the dichromate oxidation method. Total N was measured using the Kjeldahl method. Available P was extracted using the double acid method followed by the molybdenum blue method. Available K was extracted with 1.0 M ammonium acetate and determined by flame photometry (FP640; Shanghai, China).

#### Vegetation sampling

A vegetation survey was conducted at each quadrat in July 2011. The coverage and type of vegetation were recorded in each quadrat. The percentage of total plant cover was obtained, using the quadrat estimation methods^9^. The height of the tallest (longest) stem and crown width along the long and perpendicular axis were recorded for each plant.

### Statistical analysis

The Margalef species richness index (M) is defined as

1 $${\rm{M}}=({\rm{S}}-1)/\,\mathrm{ln}\,{\rm{N}}$$

where S is the number of species, and N is the total number of plants recorded in each quadrat. The Shannon-Wiener diversity index (H) was calculated to interpret species-composition change among sandy land types and sand-protecting barriers. H is defined as

2 $${\rm{H}}=-\sum _{{\rm{i}}=1}^{{\rm{S}}}({\rm{Pi}}\cdot \,\mathrm{ln}\,{\rm{Pi}})$$

where *P*_*i*_ is the relative abundance of species *i* based on its total number.

A one-way analysis of variance (ANOVA) was used to analyze the difference between mean values, and Duncan’s multiple range tests were selected to compare the means, calculated at p < 0.05 level. SPSS 20.0 software was used for these analyses.

Cluster and ordination (redundancy analysis [RDA]) analyses were conducted to analyze the similarities or differences among the different sandy land types in terms of the composition and abundance of plant species. M, vegetation coverage, and the average crown width of each species were processed by CANOCO 4.5 using the environment data (N, P, K *et al*.) for RDA with Monte Carlo permutation test.

## Results

### Vegetation composition and distribution on sandy land types

**Figure 2:**
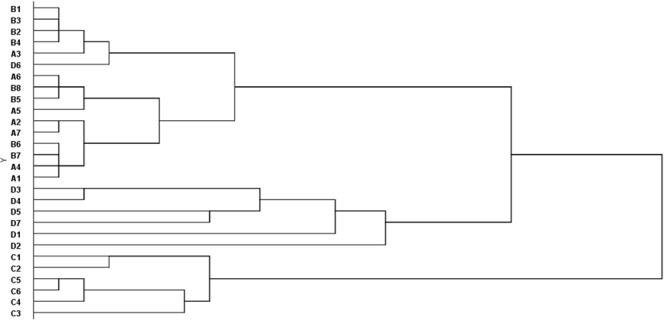
Dendrogram showing census grouping based on their species composition, abundance and vegetation coverage.

A total of 14 species were found in different sandy land types (Table 4). Five of them (*A. sphaerocephala*, *H. scoparium*, *O. sericopetala*, *A. wellbyi* and *O. thoroldii*.) were present in all four land types, but their crown width and number were different. The dendrogram (Fig. 2) obtained from the cluster analysis shows three major groups (1, 2, and 3). Group 1 primarily composes of low sand belt and sandy gravel ground where dune height and density are less than 0.1 m and 0.5, respectively. Group 2 includes all samples from the moving sandy land on the valley-slope and the dune density is more than 0.8. In group 3, the samples are from the barchan dune with the dune height and density of 2–5 m and 60–70%, respectively.

**Table 4:** Species found in the types studied. A is low sand belt, B is sandy gravel ground, C is barchan dune, D is moving sandy land on valley-slope. Crosses indicate the presence of this species in the sandy land type.

Species	Sandy land types			
	A	B	C	D
*A. sphaerocephala*	**×**	**×**	**×**	**×**
*H. scoparium*	**×**	**×**	**×**	**×**
*Hedysarum fruticosum* var. *mongolicum*	**×**	**×**		**×**
*S. moorcroftiana*	**×**		**×**	**×**
*O. sericopetala*	**×**	**×**	**×**	**×**
*A. wellbyi*	**×**	**×**	**×**	**×**
*A. frutescens*			**×**	
*A. younghusbandii*			**×**	
*H. gouldii*				**×**
*C. arborescens*				**×**
*A. tsangpoensis*				**×**
*C. minus*				**×**
*A. squarrosum*	**×**	**×**		**×**
*O. thoroldii*	**×**	**×**	**×**	**×**

**Figure 3:**
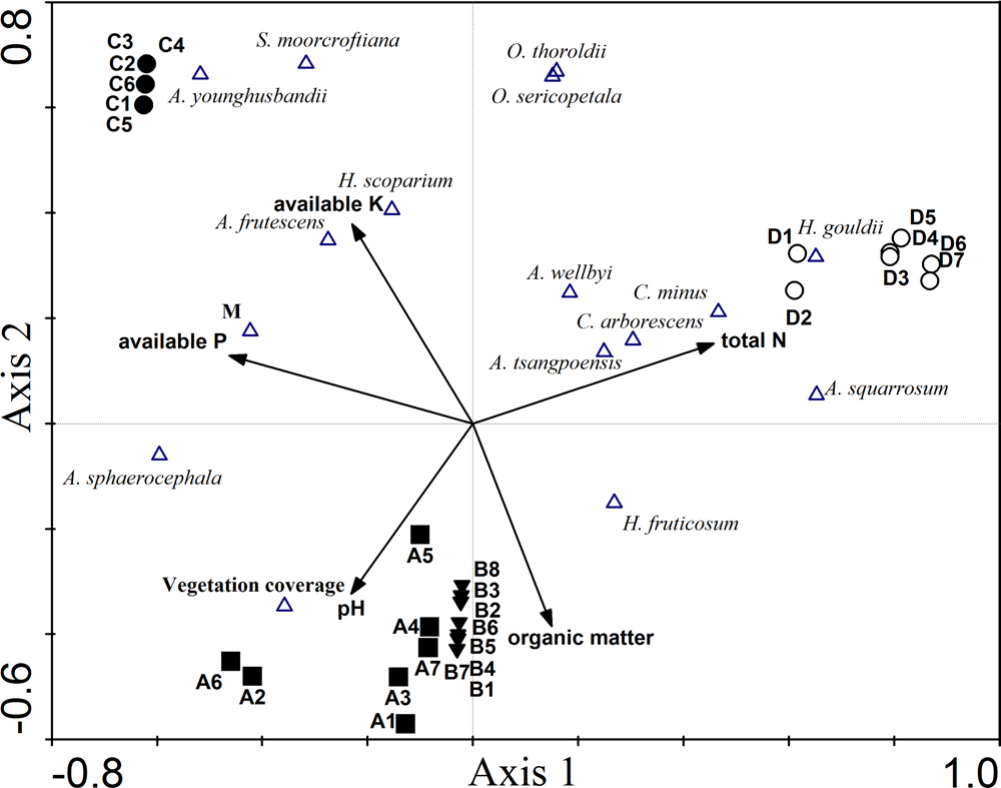
RDA of census based on their Margalef species richness index (M), vegetation coverage and the average crown width of each species, combining with various environmental factors. The Monte Carlo Permutation Test (p < 0.05): the species-environment correlation values are 0.96 and 0.94 for Axis 1 and Axis 2, respectively. Black squares are sample (**A**), black triangles are sample (**B**), black circles are sample (**C**), white circles are sample (**D**). White triangles are Margalef species richness index (M), vegetation coverage and each species. Black arrows represent environmental factors.

From RDA, axis 1, 2, 3 and 4 explain 49.1% of the sample variability (axis 1 = 24.9%, axis 2 = 19.7%, axis 3 = 2.7%, axis 4 = 1.8%). In Fig. 3 where axes 1 and 2 are drawn, three groups mentioned earlier also can be distinguished according to the different types of sandy land topography. For environmental variables, in group 1, especially the sandy gravel ground, the pH and organic matter are higher than those in other groups. Group 2 has the highest content of total N, while the available P and K are the highest in group 3. The vegetation coverage of group 1 and 3 are higher than that of group 2, while group 3 has the highest species richness. The crown widths of *S. moorcroftiana*, *A. younghusbandii*, *H. scoparium* and *Atraphaxis frutescens* have the highest positive correlation with available K and they grow well in group 3. Similarly, the crown widths of *Heteropappus gouldii*, *Ceratostigma minus*, *Calligonum arborescens*, *A. wellbyi*, *Aristida tsangpoensis* and *Agriophyllum squarrosum* have the highest positive correlation with total N and are greater in group 2 than in other groups.

**Figure 4:**
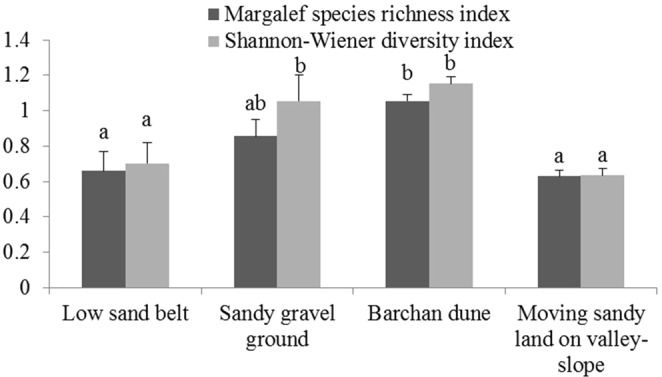
Margalef species richness index and Shannon-Wiener diversity index in different sand dune topographies. Vertical T bars indicate standard error. For the same index, the same lower-case letter shows that there is no significant difference among different sand dune topography.

As with the RDA analysis, the Margalef species richness index of the barchan dune is higher than other sand dune types and the moving sandy land on the valley-slope is the lowest. The Shannon-Wiener diversity index indicates that diversity is greater in the sandy gravel ground and barchan dune than other types (Fig. 4).

### Vegetation composition and distribution related to sand-protecting barriers

**Figure 5:**
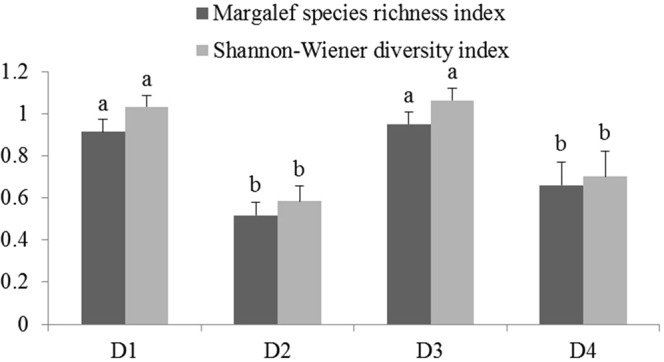
Margalef species richness index and Shannon-Wiener diversity index in different sand-protecting barriers. Vertical T bars indicate standard error. For the same index, the same lower-case letter shows that there is no significant difference among different sand-protecting barriers.

The average crown width in sites with different protecting barriers shows that the plant sizes of *A. sphaerocephala* and *A. wellbyi* are larger than other species (Table 5). *A. sphaerocephala* in D3 is the smallest, and *A. wellbyi* is the largest in D4. Various species have their own growth conditions. However, the Margalef species richness index indicates that the species richness and the Shannon-Wiener diversity index of D1 and D3 are significantly higher than that of D2 and D4 (Fig. 5).

**Table 5:** Average crown width of each species in different sand-protecting barriers. D1 is sand-protecting barrier of straw which is near the river, D2 is sand-protecting barrier of straw which is close to the road, D3 is plastic and stone checkerboard barriers, D4 is blank control.

Species	The average crown width (Length × Width)/cm			
	D1	D2	D3	D4
*A. sphaerocephala*	67.3 × 60.7	52.8 × 44.3	17.0 × 13.9	71.8 × 60.6
*H. scoparium*	18.8 × 15.5	15.5 × 9.0	15.9 × 12.1	32.3 × 23.7
*H. mongolicum*	15.0 × 12.5	—	16.1 × 13.7	22.0 × 14.0
*S. moorcroftiana*	8.3 × 5.6	66.7 × 60.3	23.0 × 21.3	6.4 × 4.6
*O. sericopetala*	17.4 × 16.1	31.0 × 24.3	17.4 × 13.4	2.0 × 2.0
*A. wellbyi*	47.6 × 38.7	68.3 × 63.2	69.1 × 58.3	97.4 × 70.8
*A. squarrosum*	3.9 × 3.5	3.5 × 3.1	2.2 × 1.6	2.3 × 1.5
*O. thoroldii*	13.5 × 11.0	7.7 × 6.7	10.0 × 7.7	8.5 × 5.5

## Discussion

### Impact of sandy land topography on revegetation

Environmental conditions vary with elevation, which might influence phenotypic plasticity and selection pressures, leading to local adaptation^36,37^. This study shows that the sandy land types have important impacts on vegetation recovery (Fig. 2). Three types can be identified based on the cluster and the redundancy analyses (Fig. 3). The growth of species was different on various sandy land topography. In this study, *A. sphaerocephala* in the low sand belt and sandy gravel ground, *A. younghusbandii* in the barchan dune, and *H. gouldii* in the moving sandy land on the valley-slope have the positive response to the topography. These species could be considered as the pioneer plants for revegetation on these sandy land types, respectively.

Topography is a primary driver of natural vegetation cover within agricultural mosaics in Turkey^38^. In addition to species growth, the differences in vegetation coverage might also be associated with topographical differences. We found that vegetation coverage increased due to the existence of a large number of plants, although the crown width was the smallest in the low sand belt and sandy gravel ground. However, it is worth exploring the phenomenon why vegetation crown height is higher, but the total percent vegetation is lower and whether the micro-topography of the slope leads to vegetation coverage decreasing in the moving sandy land on valley-slope. In addition, although the low sand belt and sandy gravel ground are the same type based on elevation, they have different Shannon-Wiener diversity index values. The difference in Shannon-Wiener index value could be caused by the gravel present on the sandy gravel ground, which has a similar impact of the stone checkerboard barriers.

### Revegetation effectiveness of different sand-protecting barriers

Earlier studies have examined whether recovery methods improve vegetation for a certain period^20^. However, the rate and intensity of change from a degraded to a more desirable community are different for different species. We found that the growth of dominant species, such as *A. Sphaerocephala* and *A. wellbyi*, decreased, while the non-dominant species increased slightly at the experimental plot compared with the untreated (D4). As Dietrich *et al*. suggested, recovery measures themselves can mask recovery effects and disturb the ecosystem^39–41^. The Shannon-Wiener diversity index is one of the indicators representing the degree of recovery of degraded lands^42^. In this study, this index value at the experimental plot is remarkably higher than the untreated plot, as found in other similar studies, except for the plot of straw barriers (D2) that has a similar index value to the control plot. Both D1 and D2 employed the straw barrier, but D1 was near the river and D2 was close to the road. Thus, the difference between D1 and D2 may be caused by human activities close to the road.

**Figure 6:**
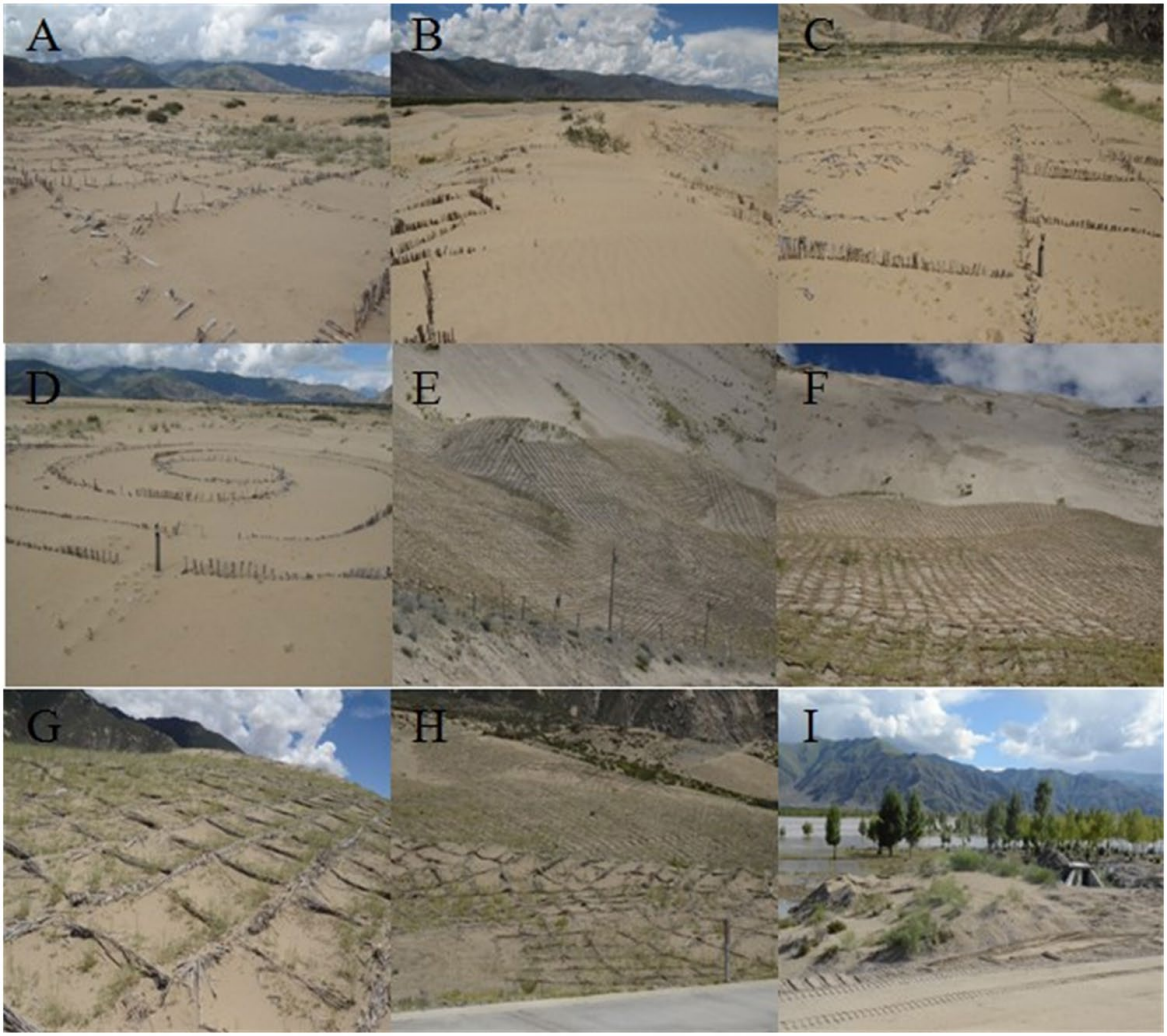
Status of revegetation in the middle reaches of Yarlung Zangbo River Basin in July 2016. (**A**–**D**) are the sand barrier of straw by local residents and government; (**E**–**H**) are the grass checkerboard barriers by local residents and government; I is *populus* and *u. pumila* plantation planted by local forestry bureau.

**Figure 7:**
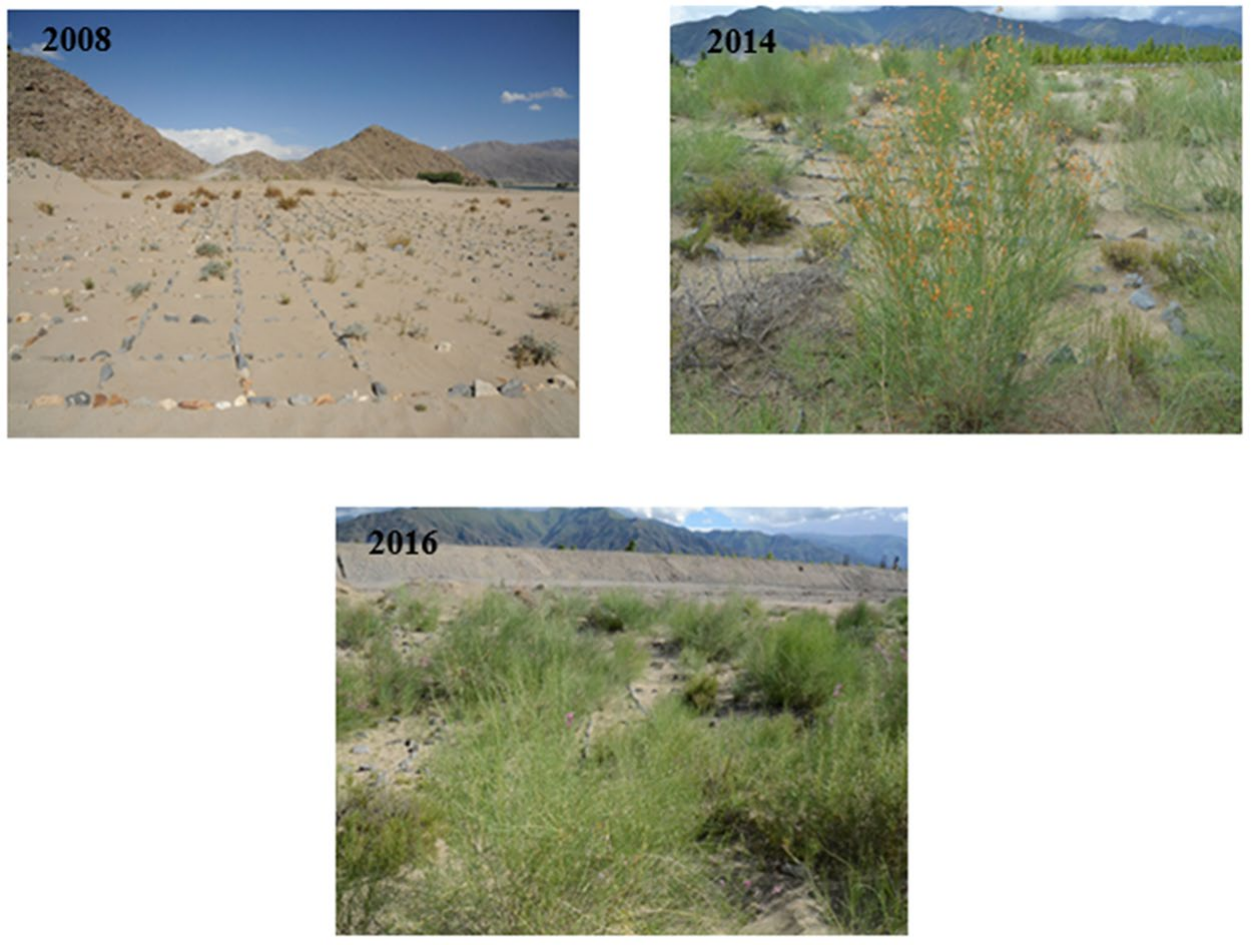
Vegetation growth on moving sandy land on the flood plain in Shannan Wide Valley from 2008 to 2016. The upper right corner is the year of photo.

Long-term planning and monitoring activities are essential for evaluating the effectiveness of revegetation methods^43^. In this study, an investigation was conducted to understand the condition of revegetation of Yarlung Zangbo River Basin in Tibet in July 2016 (Fig. 6). We also conducted random sampling to determine the state of revegetation in some of the experimental areas over time (Fig. 7). There is still a large part of the desertification land which is difficult to restore, especially the steep moving sandy land on the valley-slope. In the process of revegetation, local residents and government have made great effort. The sand-protecting barriers including grass checkerboard barriers and sand barriers of straw were laid to fix sand and protect plants. We also find that the region of grass pane achieved initial success (Fig. 6E–H), but the sand barrier of straw failed (Fig. 6A–D). The width and thickness of the straw paving might be a cause of failure. In addition, the planting of trees promotes revegetation faster than livestock exclusion and planting shrubs^21^. The local forestry bureau has established *Populus alba* var. *pyramidalis* and *u. pumila* plantations alongside our experimental area in moving sandy land on the flood plain of Shannan Wide Valley and achieved great results, although the extent of the plantations is small (Fig. 6I). We could also try to cultivate a non-native forest in above experimental area in a later study. Finally, we got scientific measures to optimize manpower and resources by comparing the recovery effect of local residents and government with the recovery effect of this research. (Fig. 7).

### Deficiencies and suggestions for future revegetation

While this study has high potential, more effort is needed to investigate the full extent of possible recovery results. Moreover, the harsh environment on the Tibetan Plateau make it challenging for field work. The terrestrial LiDAR (light detection and ranging) technology should be considered to monitor this continuously changing system. Through the investigation of different types of sandy land topography on revegetation, and the impact of sand-protecting barriers in moving sandy land on floodplain, we identified the effective method of revegetation on moving sandy land on river bank and valley-slope. We believe that our results would provide basic and critical information for the research of sustainable and dynamic revegetation in the alpine ecoregion. In the initial revegetation period, describing species composition and morphological differences will provide a foundation for how time dynamics and climate change might affect vegetation recovery. Combined with other studies, such as Li *et al*.^44^ and Zhang *et al*.^26^ suggesting that supplemental seeding would expedite recovery, this study provides a reference for the study of revegetation in the whole Yarlung Zangbo River basin and even all of Tibet.

## Conclusions

In this paper, we analyzed the differences in revegetation on four sandy land types that can be divided into three topographic types. A total of 14 species were tested in all these types and a significant difference was observed among the growths of species in each type. Specifically, *A. sphaerocephala* grew the best on the low sand belt and sandy gravel ground dune types, *A. younghusbandii* favors the barchan dune, and *H. gouldii* grew the best on the moving sandy land on the valley-slope. These species could be considered as the pioneer plants for revegetation on these land types.

The effectiveness of revegetation is different among different sand-protecting barriers of the moving sandy land on floodplain. The growth of dominant species, such as *A. Sphaerocephala*, decreased, while the non-dominant species increased slightly after building sand-protecting barriers in the early stage of recovery. Sand-protecting barriers played important roles on revegetation. The stone checkerboard barriers improve plant diversity, while the straw barrier is more beneficial to the growth of individual species. This study provides an ecological foundation and suggestions for implementing and developing suitable revegetation strategies on sandy land on the Tibetan Plateau.

## Supplementary information

Supplementary information

## Data Availability

The datasets generated during and/or analyzed during the current study are available from the corresponding author on reasonable request.

## References

[1] Chen X, Duan Z, Tan M (2016). Restoration Affect Soil Organic Carbon and Nutrients in Different Particle-size Fractions. Land Degradation & Development..

[2] Shen W, Li H, Sun M, Jiang J (2012). Dynamics of aeolian sandy land in the Yarlung Zangbo River basin of Tibet, China from 1975 to 2008. Global and Planetary Change..

[3] Safriel U (2017). Land Degradation Neutrality (LDN) in drylands and beyond–where has it come from and where does it go. Silva Fennica..

[4] Zang Y, Gong W, Xie H, Liu B, Chen H (2015). Chemical sand stabilization: A review of material, mechanism, and problems. Environmental Technology Reviews..

[5] Hill J, Stellmes M, Udelhoven T, Roder A, Sommer S (2008). Mediterranean desertification and land degradation: mapping related land use change syndromes based on satellite observations. Global and Planetary Change..

[6] Onate JJ, Begona P (2005). Policy impact on desertification: stakeholders’ perceptions in southeast Spain. Land use policy..

[7] Salvati L, Sofia B (2011). Land sensitivity to desertification across Italy: past, present, and future. Applied Geography..

[8] Wang F, Pan X, Wang D, Shen C, Lu Q (2013). Combating desertification in China: past, present and future. Land Use Policy..

[9] Ebrahimi M, Mohammadi F, Fakhireh A, Bameri A (2017). Effect of *Haloxylon* spp. with Different Age Classes on Vegetation Cover and Soil Properties in an Arid Desert Steppe of Iran. Pedosphere.

[10] Duan J, Litwiller E, Choi SH, Pinnau I (2014). Evaluation of sodium lignin sulfonate as draw solute in forward osmosis for desert restoration. Journal of Membrane Science..

[11] Amiraslani F, Deirdre D (2011). Combating desertification in Iran over the last 50 years: an overview of changing approaches. Journal of Environmental Management..

[12] Choi YD (2004). Theories for ecological restoration in changing environment: toward ‘futuristic’ restoration. Ecological Research..

[13] Benli L, Qu J, Kang S (2016). Response of dune activity on the Tibetan Plateau to near future climate change. Climate Research..

[14] State Forestry Administration, P. R. China. The Bulletin of Desertification and Sandification State of China (2015).

[15] Mueller EN, Wainwright J, Parsons AJ (2007). The stability of vegetation boundaries and the propagation of desertification in the American Southwest: A modelling approach. Ecological Modelling..

[16] Becerril-Pina R, Mastachi-Loza CA, Gonzalez-Sosa E, Diaz-Delgado C, Ba KM (2015). Assessing desertification risk in the semi-arid highlands of central Mexico. Journal of Arid Environments..

[17] Sun H, Zheng D, Yao TD, Zhang Y (2012). Protection and construction of the national ecological security shelter zone on Tibetan Plateau. Acta Geographica Sinica..

[18] Li H (2016). Human Impact on Vegetation Dynamics around Lhasa, Southern Tibetan Plateau, China. Sustainability..

[19] Shao Q (2016). Assessment on the effects of the first-stage ecological conservation and restoration project in Sanjiangyuan region. Acta Geographica Sinica..

[20] Miyasaka T, Okuro T, Miyamori E, Zhao X, Takeuchi K (2014). Effects of different restoration measures and sand dune topography on short- and long-term vegetation restoration in northeast China. Journal of Arid Environments..

[21] Miao C, Li X, Jia M, Han X, Jiang D (2016). Spatial structure and species composition of soil seed banks in moving sand dune systems of northeast China. Journal of forestry research..

[22] Wang SK (2016). Responses of soil fungal community to the sandy grassland restoration in Horqin Sandy Land, northern China. Environmental monitoring and assessment..

[23] WallisDeVries MF, Bobbink R (2017). Nitrogen deposition impacts on biodiversity in terrestrial ecosystems: Mechanisms and perspectives for restoration. Biological Conservation..

[24] Li YF (2017). Impacts of artificially planted vegetation on the ecological restoration of movable sand dunes in the Mugetan Desert, northeastern Qinghai-Tibet Plateau. International Journal of Sediment Research..

[25] Zhao HL (2007). Shrub facilitation of desert land restoration in the Horqin Sand Land of Inner Mongolia. Ecological engineering..

[26] Zhang J, Zhao H, Zhang T, Zhao X, Drake S (2005). Community succession along a chronosequence of vegetation restoration on sand dunes in Horqin Sandy Land. Journal of Arid Environments..

[27] Zuo XA (2010). Spatial pattern and heterogeneity of soil organic carbon and nitrogen in sand dunes related to vegetation change and geomorphic position in Horqin Sandy Land, Northern China. Environmental monitoring and assessment..

[28] Cao C (2016). Assessment of the effects of phytogenic nebkhas on soil nutrient accumulation and soil microbiological property improvement in semi-arid sandy land. Ecological Engineering..

[29] Liu R, Zhu F, Steinberger Y (2016). Ground-active arthropod responses to rainfall-induced dune microhabitats in a desertified steppe ecosystem, China. Journal of Arid Land..

[30] Ma W, Wang X, Zhou N, Jiao L (2017). Relative importance of climate factors and human activities in impacting vegetation dynamics during 2000–2015 in the Otindag Sandy Land, northern China. Journal of Arid Land..

[31] Luo Y, Zhao X, Li Y, Wang T (2017). Effects of foliage litter of a pioneer shrub (*Artemisia halodendron*) on germination from the soil seedbank in a semi-arid sandy grassland in China. Journal of Plant Research..

[32] Sun B (2017). Grassland degradation and restoration monitoring and driving forces analysis based on long time-series remote sensing data in Xilin Gol League. Acta Ecologica Sinica..

[33] Li H (2015). Elevation-dependent vegetation greening of the Yarlung Zangbo River basin in the southern Tibetan Plateau, 1999–2013. Remote Sensing..

[34] Li H (2013). Spatio-temporal variability of soil moisture and its effect on vegetation in a desertified aeolian riparian ecotone on the Tibetan Plateau, China. Journal of Hydrology..

[35] Overton MF, Pratikto WA, Lu JC, Fisher JS (1994). Laboratory investigation of dune erosion as a function of sand grain size and dune density. Coastal Engineering..

[36] Haider S, Kueffer C, Edwards PJ, Alexander JM (2012). Genetically based differentiation in growth of multiple non-native plant species along a steep environmental gradient. Oecologia..

[37] Frei ER, Ghazoul J, Matter P, Heggli M, Pluess AR (2014). Plant population differentiation and climate change: responses of grassland species along an elevational gradient. Global change biology..

[38] Pekin BK (2016). Anthropogenic and topographic correlates of natural vegetation cover within agricultural landscape mosaics in Turkey. Land Use Policy..

[39] Dietrich AL, Nilsson C, Jansson R (2015). Restoration effects on germination and survival of plants in the riparian zone: a phytometer study. Plant ecology..

[40] Tullos DD, Penrose DL, Jennings GD, Gregory Cope W (2009). Analysis of functional traits in reconfigured channels: implications for the bioassessment and disturbance of river restoration. Journal of the North American Benthological Society..

[41] Catford JA (2012). The intermediate disturbance hypothesis and plant invasions: Implications for species richness and management. Perspectives in Plant Ecology, Evolution and Systematics..

[42] Miao R (2015). Effectiveness of shrub planting and grazing exclusion on degraded sandy grassland restoration in Horqin sandy land in Inner Mongolia. Ecological Engineering..

[43] Fill JM, Forsyth GG, Kritzinger-Klopper S, Le Maitre DC, van Wilgen BW (2017). An assessment of the effectiveness of a long-term ecosystem restoration project in a fynbos shrubland catchment in South Africa. Journal of Environmental Management..

[44] Li Y, Cui J, Zhao X, Zhao H (2004). Floristic composition of vegetation and the soil seed bank in different types of dunes of Kerqin steppe. Arid Land Research and Management..

