# Supplementary material for: Effect of topography and protecting barriers on revegetation of sandy land, Southern Tibetan Plateau
Source: Sci Rep. 2019 Apr 24;9:6501. doi: 10.1038/s41598-019-43034-8 (PMC6482179; doi:10.1038/s41598-019-43034-8)
Supplement: Supplementary file 1 — Supplementary information [file 41598_2019_43034_MOESM1_ESM.pdf]

Effect of topography and protecting barriers on revegetation of sandy land, Southern Tibetan Plateau

Chengrui Liao[1, 3, 4], Beichen Liu[1], Yannan Xu[1, 3*], Yingkui Li[5], Haidong Li[2,*]

Supplementary table 1 Environment data in different sandy land types

Identifier	Location	pH	Organic matter /g • kg^{-1}	Total nitrogen / g • kg^{-1}	Available phosphorus /mg • kg^{-1}	Available potassium /mg • kg^{-1}
A	Moving sandy land on flood plain	8.76	1.49	0.09	1.97	26.83
B	Moving sandy land on flood plain	9.07	1.87	0.11	1.76	28.29
C	Moving sandy land on river bank	8.23	0.25	0.03	2.69	40.00
D	Moving sandy land on valley-slope	7.59	1.05	1.04	1.30	29.89

A: Low sand belt; B: Sandy gravel ground; C: Barchan dune; D: Moving sandy land on valley-slope.
